# Profibrotic predictive toxicology in the lung

**DOI:** 10.3389/fphar.2026.1766054

**Published:** 2026-02-18

**Authors:** Pooja Singh, Rajesh Sinha, Veena B. Antony

**Affiliations:** Division of Pulmonary, Allergy and Critical Care Medicine, Department of Medicine, Heersink School of Medicine, University of Alabama at Birmingham, Birmingham, AL, United States

**Keywords:** 3D culturemodel, chemical induced toxicity, environmental toxicants, predictive toxicology, pulmonary toxicity

## Abstract

**Introduction:**

Fibrosis in the gossamer alveolar capillary membranes of the lung can lead to abnormalities in gas exchange, hypoxemia and death of the individual. These interstitial lung diseases (ILDs) of known or yet undefined etiologies (such as Idiopathic pulmonary fibrosis) highlight the need for predictive, physiologically relevant models for toxicity studies. Three-dimensional (3D) lung organoids derived from animal cells provide an advanced platform that replicates the structural and cellular complexity of lung tissue while reducing whole-animal use.

**Methods:**

Mouse lung organoids (MiLO) were used to evaluate pulmonary toxicity caused by environmental toxicants and pharmacologic agents. MiLO were generated from perfused, minced mouse lungs that were digested with collagenase, filtered, depleted of red blood cells, and embedded in Matrigel. Organoids were stained for lineage markers to characterize cellular diversity, including SPC, α-SMA, CD31, F4/80 and ECM proteins collagen I and fibronectin. Gene expression in MiLO and native lung tissue was compared for fibrosis- and viability-related markers. A well-characterized mouse model of cadmium induced lung fibrosis was used as an in vivo benchmark to assess α-SMA expression, airway resistance to methacholine, hydroxyproline content, malondialdehyde levels (MDA), and superoxide dismutase (SOD) activity. For drug-induced fibrosis modeling, cell viability assays defined 20% inhibitory concentrations of nitrofurantoin (NF, 5 μM) and amiodarone (AD, 20 μM), which were then used to treat MiLO for assessment of MDA, invasion area on collagen-coated plates, and expression of fibrotic and signaling markers.

**Results:**

MiLOs faithfully recapitulated native lung architecture, extracellular matrix composition, and fibrosis-related gene expression profiles. In vivo cadmium exposure increased α-SMA expression, airway resistance, collagen content, and malondialdehyde (MDA) levels, while reducing superoxide dismutase (SOD) activity. Consistently, Cd- treated MiLOs exhibited increases in COL1A1 deposition, cellular invasion, hydroxyproline content, and oxidative stress. Exposure to nitrofurantoin (NF) or amiodarone (AD) elevated MDA, enhanced invasion, and upregulated fibrogenic and signaling genes including Tgfb1, Col1a1, Acta2, Akt1, Nfkb1, and Mmp9. Environmental toxicant (Cd) and drug (AD or NF) treatments drove the development of hallmark fibrotic features in lung organoids, characterized by increased collagen deposition, oxidative stress, and profibrotic gene activation.

**Conclusions:**

These findings demonstrate that mouse lung organoids effectively recapitulate key molecular and pathological aspects of drug- and toxin-induced pulmonary fibrosis and represent a powerful model for mechanistic investigation and preclinical screening of compounds with potential pro-fibrotic effects.

## Introduction

Interstitial lung diseases (ILDs) are a diverse group of disorders marked by inflammation and fibrosis of the lung interstitium (Althobiani et al., 2024). The incidence and prevalence of ILDs are rising globally, leading to increased morbidity and early mortality (Althobiani et al., 2024; Schwaiblmair et al., 2012). Certain ILD subtypes are associated with identifiable environmental or occupational exposures, such as organic antigens, asbestos, or mineral dust, while idiopathic forms like idiopathic pulmonary fibrosis (IPF) often involve prior inhalational exposures, including vapors, gases, dust, and fumes. A meta-analysis revealed that several exposures were significantly associated with increased ILD risk-smoking (odds ratio [OR] 1.69), organic exposures (OR 1.56), metals (OR 1.52), dust (OR 1.45), and asbestos (OR 1.53). Air pollutants such as nitrogen dioxide (NO_2_), ozone (O_3_), and fine particulate matter (PM2.5) aggravate oxidative stress and epithelial injury, precipitating both disease onset and acute exacerbations (Lee et al., 2025). Chronic exposure to such toxins enhances fibrogenic cytokine release, including IL-13, IL-4, and osteopontin, and promotes epithelial-mesenchymal transition and fibroblast activation. Emerging evidence links long-term PM2.5 exposure to a 2.5-fold increase in acute IPF exacerbation risk and to accelerated telomere attrition, emphasizing the mechanistic interplay between environmental stressors, genetic vulnerability, and age-related decline (Wang et al., 2024).

Experimental animal models are invaluable in elucidating disease mechanisms and assessing the effects of environmental exposures on lung injury and fibrosis. For instance, mice share approximately 80% of their genome with humans, making them valuable models for studying human diseases (Breschi et al., 2017). These similarities allow animals to develop conditions that closely resemble human diseases, enabling researchers to investigate disease mechanisms and potential treatments. Another significant advantage of animal models is their shorter lifespan compared to humans, which allows scientists to study the progression of age-related diseases within a compressed timeframe (Harper et al., 2006; Mitchell et al., 2015). This enables researchers to observe the full course of a disease and its effects over a lifetime more efficiently than would be possible in human studies. However, they present ethical concerns and biological differences that can limit translational relevance. To address these challenges, *in vitro* 3D models derived from animal lung tissue are increasingly recognized as critical tools for environmental toxicity studies (Edmondson et al., 2014). These 3D models better replicate the structural and cellular complexity of the lung microenvironment compared to traditional two-dimensional cultures (Edmondson et al., 2014; Wang et al., 2025). They preserve crucial cell–cell and cell–matrix interactions, oxygen and nutrient gradients, and physiological responses to toxicants, enabling more accurate assessment of inhaled pollutants’ effects on lung tissue (Edmondson et al., 2014). Importantly, 3D models reduce reliance on animal testing, aligning with the 3R principles by supporting Replacement and Refinement strategies while facilitating mechanistic toxicology research (Lee et al., 2020).

To improve predictive toxicology for pulmonary fibrosis, we prepared animal tissue-derived 3D lung cell culture platform (MiLO) that more accurately mimics lung structure, function, and cellular interactions than traditional models. These mouse lung derived organoids (MiLO) provide enhanced modeling of complex cellular environments, enabling better assessment of drug-induced pulmonary toxicity. They are valuable for evaluating fibrosis triggered by environmental toxins and pharmaceutical agents, advancing mechanistic insight and toxicity prediction in drug development.

## Methods

### Lung tissue samples

6–8 weeks-old C57BL/6 mice lung tissue was harvested as per institutional guidelines at UAB. single-cell suspension preparation involved the following steps. Mice lungs were perfused with 2.5 mL PBS and washed with cold PBS solution. Bronchi were carefully avoided, and tissue was sectioned and minced before adding 5 mL of collagenase A. This was incubated for 30 min at 37 °C with gentle agitation every 10 min. After the incubation, 10 mL of PBS was added and suspension were passed through 100µ cell strainer. The filtered suspension was centrifuged for 5 min at 300 g and washed with complete DMEM consisting of 10% FBS, penicillin and streptomycin, and Glutamax. The cell pellet was then resuspended in ACK lysis buffer to lyse red blood cells and was incubated at room temperature for 5 min with occasional shaking. The reaction was stopped by diluting the ACK lysis buffer with 30 mL of PBS, and the cells were centrifuged at 300 *g* for 5 min. The supernatant was removed carefully, and the pellet was resuspended in minimal essential media (MEM, Gibco) supplemented with non-essential amino acids (Gibco) and 1% antibiotic-anti-mycotic solution (Gibco).

### Cell viability assay

Cell suspension obtained was assessed for cell viability upon drug treatment. Drug concentration leading to 80% viability of cells was selected for drug exposure related experiments. Cell viability assay was performed using MTT based Cell Proliferation Kit I, following the manufacturer’s protocol (11 465 007 001; Roche). Briefly, Cells were seeded in 96-well flat-bottom plates and treated as indicated for 24 h. MTT labeling reagent was then added to each well (final concentration 0.5 mg/mL), and plates were incubated for 4 h at 37 °C to allow formation of intracellular formazan crystals. The solubilization solution was then added and incubation was continued until complete dissolution of the formazan, after which absorbance was recorded at 570 nm using a microplate reader (Synergy HTX, Biotek). Cell viability for each condition was calculated after blank subtraction as 100 × [(A_treated_−A_blank_)/(A_control_−A_blank_)], with untreated controls defined as 100% viability, and drug concentrations displaying approximately 80% viability (i.e., 20% inhibition) were identified based on this calculation.

### Preparation of 3D mice lung organoids and treatment

Anti-adherence rinsing solution was added to each well of a 96-well U-bottom plate, to ensure organoids were free floating and accessible to harvest from the wells without trypsinization. The plates were left at room temperature in a sterile laminar hood for 30 min. Solution was collected, wells were washed with PBS (twice) and air dried. These coated plates were then used for the preparation of MiLO. MiLO were prepared from cell suspension obtained from lung tissue. The total cell pellet was resuspended in 2 mL complete MEM medium. Cells were stained with trypan blue and counted to check for cell density and viability. Cell suspension was mixed with corning organoid matrigel matrix (356255, Corning, United States) in 1:1 ratio. 8,000–10,000 cells (depending upon total number of cells and assays) were added per well in the U-bottom 96 well plates. Plates were incubated for 30 min at 37 °C in CO_2_ (5%) incubator. Corning Matrigel Matrix for Organoid Culture is a basement membrane extract from EHS mouse sarcoma, rich in ECM proteins like laminin, collagen IV, entactin, heparan sulfate proteoglycans, and growth factors. Once matrigel solidified complete media was added to each well and plates were incubated for 3 days and fresh media was replaced every other day. Cells take spheroid shape in 2 days. The resulting 3D mice lung organoids (MiLO) were treated with low dose of cadmium chloride (CdCl_2_, 10 μM; CAS 10108-64-2, Sigma-Aldrich), amiodarone (AD, 20 μM; CAS J6045606, Thermo Scientific Chemicals) or nitrofurantoin (NF, 5 μM; CAS B2407914, Thermo Scientific Chemicals) for 24 h. When treatment was complete, MiLO were fixed and paraffinized to section to stain for fibrotic markers visualized using All-in-One Fluorescence Microscope BZ-X810 (Keyence, United States).

### Cd exposed mouse model

Animals were purchased from the Jackson Laboratory and all procedures were approved by the Institutional Animal Care and Use Committee of the University of Alabama at Birmingham. Mice (6–8 weeks old; 25–30 g) of both sexes were included, with equal numbers of females and males per group. Animals were acclimatized for at least 1 week before experimentation and then randomly allocated to treatment or control groups. Mice were treated once by intratracheal instillation with cadmium chloride (CdCl_2_; Sigma-Aldrich, CAS 10108-64-2, 0.458 mg/kg) or saline vehicle as previously described for Cd-associated mouse models of lung injury and remodeling. Lung tissues were collected on days 0, 3, 6, 9, 12, 15, 18, and 21 (n = 3 per time point per treatment group) for biochemical and histological analyses. Hydroxyproline content was quantified according to the manufacturer’s instructions (Abcam) as an indicator of collagen deposition and fibrosis. A separate cohort of mice (n = 3 per group) was assessed for respiratory mechanics at day 21 post-instillation. Following anesthesia, airway resistance (Rrs) was measured using the single-compartment model as previously described (Li et al., 2021; Li et al., 2022).

### Measurement of collagen

For collagen quantification, the right lung from mice was removed, weighed, and homogenized. Collagen content in mouse lung tissue and MiLO was assessed by the Hydroxyproline assay (ab222941, Abcam) following the manufacturer’s instructions. The soluble collagen levels in supernatants from Cd treatment cells were also evaluated.

### Immunohistochemistry of MiLO

MiLO were washed and fixed in formalin for 24 h at room temperature. Formalin was pipetted out, and 100 μL of warmed (at 80 °C) histogel (Corning, United States) was added to the MiLO. The histogel- MiLO suspension was refrigerated until the gel became firm. The gel was taken out from the tube with the help of a spatula and kept in a cassette. The cassette was incubated in formalin solution overnight. These cassettes were processed for paraffin sectioning to obtain 5 µ thin sections.

### Immunofluorescent staining of MiLO

After blocking in PBST (0.1% Triton X-100 in PBS) containing 3% Bovine Serum Albumin (Sigma-Aldrich) overnight at 4 °C and washing in PBST (twice for 15 min), MiLO were incubated with primary antibodies diluted in PBST on a gently rocking rotator at 4 °C for 48 h and rinsed in PBST (4 times for 30 min). When necessary, MiLO were then incubated in appropriate AlexaFluor-conjugated secondary antibodies (Molecular Probes, Life technologies) for 24 h. Cell nuclei were counterstained by DAPI (Invitrogen) diluted 1:500 in PBS for 40 min at room temperature. Immunolabeled MiLO were imaged using confocal microscopy. For paraffin sections, immunofluorescent staining was performed for SPC (ab32575, Abcam), CD31 (558737, Thermo Fisher), F4/80 (123116, Abcam), α-SMA (NB300-978, Novus Bio.), Fibronectin (FN1; ab54477, Abcam), Ki67 (sc15402, Santa Cruz), COL1A1 (ab260043, Abcam) and Ig G isotype (sc 2005, Santa Cruz). All immunofluorescence-stained slides were co-stained with DAPI for visualization of the nucleus using All-in-One Fluorescence Microscope BZ-X810 (Keyence, United States).

### RNA isolation, reverse transcription (RT), and quantitative PCR (qPCR)

Lung tissue and MiLO were processed for total RNA isolation using TRIzol reagent (Invitrogen, United States) and cDNA was synthesized to perform qRT-PCR for gene expression analysis. Briefly, the manual extraction of total RNA TRIzol reagent was added to the sample, followed by chloroform and isopropanol for RNA precipitation. Ethanol gradient washing provided total RNA, which was then dissolved in water. Concentrations were recorded and 2 µg of each RNA was mixed with cDNA synthesis master mix (1708891, iScript cDNA synthesis kit, BioRad, United States) PCR was run as per the manufacturer’s protocol. cDNA synthesized was used as a template for amplification and quantification of target genes by quantitative real-time PCR (StepONE, Applied Biosystems, United States) using TaqMan fast master mix (Thermo Fisher Scientific, United States). ß-actin was co-amplified for semi-quantitative comparison. TaqMan primer assay mix with forward and reverse sequence. Relative expression was calculated by using the 2^−ΔΔCT^ method. The reactions were performed in duplicates for each sample and mean value of 2^−ΔΔCT^ was plotted on heat map.

### Invasion assay

MiLO were collected from 96-well plates and suspended in media. Rat tail collagen type I (3.98 mg/mL, CLS354236 Corning) was mixed with neutralization solution as per the manufacturer’s instructions to prepare collagen hydrogels. MiLO were incorporated into a final collagen concentration of 2 mg/mL for media alone group without a test drug in a single well. For treatment groups, single MiLO were suspended in 100 μL of the collagen-DMEM (DMEM with 10% FBS and 1% antibiotic-antimycotic solution) solution containing Cd (10 μM) or amiodarone (20 μM) or nitrofurantoin (5 μM). The suspension was then poured into a ultra-low attachment 96-well clear flat bottom plates (corning) and gelled for 30 min at 37 °C. Fresh medium was added, and samples were imaged on Day 0. After imaging, samples were incubated at 37 °C with 5% CO_2_ for 24 h to allow cell invasion into the matrix. In total, n = 5 MiLO were seeded for each treatment. The plates were incubated at 37 °C in a CO_2_ incubator overnight. Images were captured with Keyence microscope, the invasive cells’ area was delineated and calculated (H). Additionally, the center of the MiLO was outlined and measured (R). The percentage of the total invaded area (ZOI%) was determined by (H - R)/R x 100. The percentage invasion area was calculated. The ZOI fold change was measured as the ratio of the ZOI% with treatment to the ZOI% without treatment *in vitro* with an antifibrotic drug.

### Oxidative stress analysis

Lipid peroxidation assay (MDA assay, ab233471, Abcam) and Superoxide dismutase (SOD) activity (ab65354, Abcam) were performed to estimate oxidative stress in mice lung tissue or MiLO as per the manufacturer’s protocol. Experiments were performed in triplicates.

### Statistics

Data was analyzed by one-way analysis of variance (ANOVA) followed by Dunnett multiple comparison *post hoc* test and two-way ANOVA followed by Sidak multiple comparison *post hoc* test (only if *p* < 0.05). The response rate to each treatment was calculated, and an exact 95% confidence interval around the response rate was calculated using the Clopper-Pearson method. Differences were considered statistically significant at *p* < 0.05. The data is presented as the means ± standard error unless otherwise stated.

## Results

### Characterization of cellular diversity in MiLO

**FIGURE 1:**
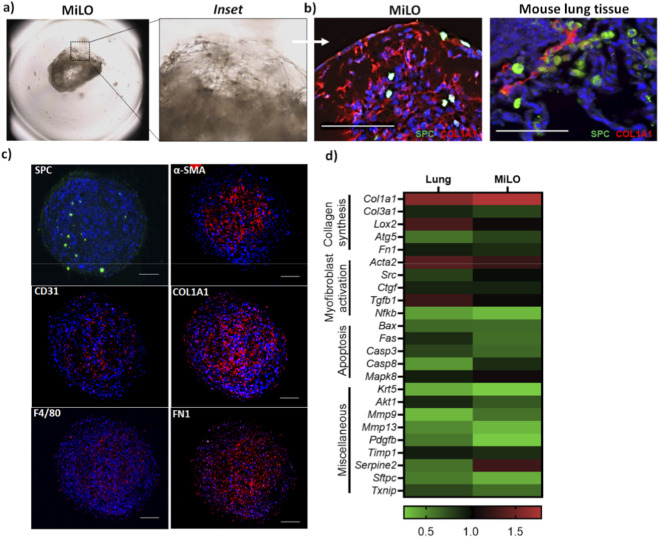
Mouse lung derived organoids (MiLO) mimic lung tissue multicellular architecture: **(a)** Brightfield microscopy of MiLO along with an inset showing cellular arrangement. **(b)** MiLO stained with SPC (ATII type cells) and collagen 1 shows similar cellular arrangement compared to mouse lung tissue section. **(c)** Different cellular markers stained in MiLO to show arrangement and presence- SPC, CD31, F4/80, α-SMA, Collagen 1 and FN1. **(d)** Gene expression analysis shown with heat map of absolute expression quantification in lung sections and MiLO.

To prepare mice lung tissue derived organoids, mice lungs were perfused, minced (excluding primary bronchi), and digested with collagenase. Cells were filtered through a 100 µm strainer, pelleted, and treated with ACK lysis buffer to remove RBCs. The cell suspension was mixed 1:1 with Matrigel, and 50 µL (0.5 × 10^4^ cells) was dispensed into each well of a round-bottom 96-well plate. After 30 min incubation at 37 °C, culture media was added, with media replacement every 2 days over a 6-day incubation period. organoids were harvested using blunt end tips in 2 mL tubes (n = 10/tube), fixed, paraffin embeded for sectioning and stained for cellular markers. These 3D organoids (Figure 1a) generated from mouse lung tissue formed multicellular structure mimicking mouse lung tissue architecture (Figure 1b). These MiLO contained various lung cell types, including epithelial type II cells expressing surfactant protein C (SPC), myofibroblasts or vascular smooth muscle cells positive for α-smooth muscle actin (α-SMA), endothelial cells labeled by CD31 and macrophages identified by F4/80 (Figure 1c). These diverse cells were embedded within extracellular matrix components such as collagen I (COL1A1) and fibronectin-I (FN1), closely replicating the native lung microenvironment and architectural organization (Figure 1c). Lung tissue and MiLO were further compared to predicting the gene expression pattern (Figure 1d). A comparable expression level was observed for fibrosis related markers such as *Tgfb1*, *Acta2*, *Col1a1*, *Col3a1*, *Ctgf*, *Lox2*, *Pdgfb* and *Fn1*, which were selected due to their critical roles in collagen synthesis, ECM remodeling, cross-linking, and fibrotic progression. To ensure the physiological relevance of the model, apoptosis-related genes including *Bax*, *Fas*, *Mapk8*, *Casp3*, and *Casp8* were analyzed to monitor programmed cell death and confirm cell viability at the time of assessment. Other genes evaluated were also evaluated here. The inclusion of basal cells expressing *Krt5*, important progenitors for airway repair, further highlights the diversity of progenitor populations. These diverse cells reside within extracellular matrix components such as collagen I and fibronectin 1, replicating the native lung microenvironment and organization. Additionally, genes involved in cell survival (*Akt1*), matrix remodeling (*Mmp9*, *Mmp13*, *Timp1*, *Serpine2*), oxidative stress (*Txnip*) (Lin et al., 2025), and inflammation regulation (*Nfkb1*) were assessed to capture the complex signaling and proteolytic landscape within the organoids (Cabral-Pacheco et al., 2020; Craig et al., 2015). Together, these genes provide insights into progenitor cell populations, matrix remodeling dynamics, antioxidant status, and epithelial cell function, thereby validating the organoid as a multi-cellular model reflecting lung tissue complexity and fibrosis-relevant pathways.

### Environmental toxicant (Cadmium) induces pro-fibrotic response in mice model

**FIGURE 2:**
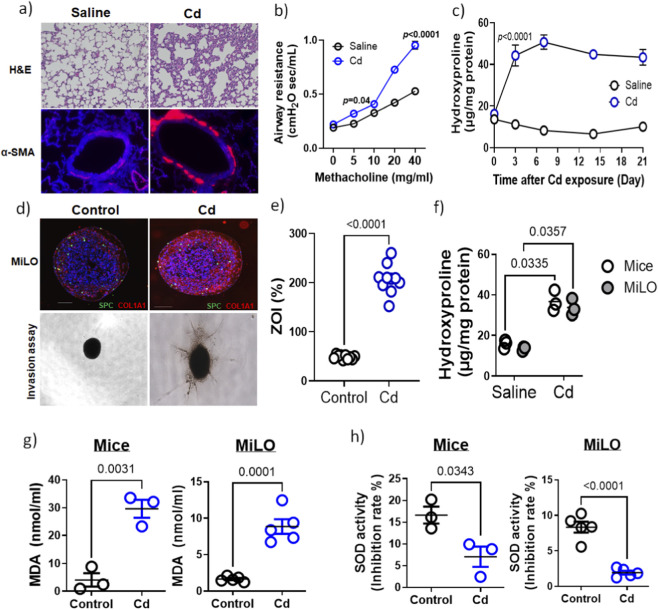
Cd-induces pulmonary toxicity with increase in pro-fibrotic markers. **(a)** Mice were exposed to Cd (0.458 mg/kg) and H&E stain control mice lung tissue (saline) shows normal histology compared to Cd exposed mice. Sections also stained for myofibroblast marker (α-SMA) staining. **(b)** Airway resistance in also increased in Cd exposed mice (n = 3). *p* < 0.01 **(c)** Hydroxyproline assay for lung tissue from mice exposed to saline or Cd at a regular interval (0, 3, 6, 9, 12, 15, 18, 21 days). **(d)** Immunofluorescence imaging of Cd (10 µM) exposed MiLO showing SPC (green) and COL1A1 (red). Invasion assay of MiLO exposed to Cd were observed using bright field microscopy. **(e)** Zone of invasion (%) was estimated for these MiLO. *p* < 0.0001. **(f)** Hydroxyproline assay for comparing collagen levels in mice lung tissue (day 21) and MiLO. Equal dry weight was used for the assay. Experiments were performed in triplicates. Cd induces oxidative stress in MiLO. Oxidative stress estimated using **(g)** MDA (nmol/mL) and **(h)** SOD activity (inhibition rate %) in lung tissue from mice (n = 3) exposed to Cd (0.458 mg/kg) and MiLO (n = 5) exposed to Cd (10 µM) for 24 h.

The pulmonary toxicity of cadmium (Cd) is highly dose- and exposure-dependent (Li et al., 2022). Our laboratory has established well-characterized mouse models demonstrating Cd-induced lung toxicity (Li et al., 2021; Li et al., 2017). Mice (n = 3) were exposed to a pre-evaluated Cd dose (0.458 mg/kg), reflecting concentrations found in human lung tissue exposed to Cd. Histological analysis revealed increased expression of α-SMA, a myofibroblast marker, in lung tissue following Cd exposure (Figure 2a). Functional assessment via methacholine challenge demonstrated a dose-dependent increase in airway resistance in Cd-treated mice compared to controls (Figure 2b), indicative of fibrosis-associated airflow obstruction. Elevated extracellular matrix deposition was quantitatively confirmed by hydroxyproline assay (Figure 2c). These results confirm that low-dose Cd exposure, relevant to environmental, occupational, and behavioral sources such as smoking, induces pulmonary fibrosis characterized by myofibroblast activation and collagen accumulation, impairing lung function. This mouse model thus provides a critical benchmark for evaluating pulmonary toxicity mechanisms and supports the translational utility of lung organoid systems in toxicology research.

### MiLO mimic *in vivo* characteristics of Cd-induced lung fibrosis

To evaluate the relevance of mouse lung organoids in toxicity assessments, we compared outcomes from a well-established Cd-induced lung fibrosis mouse model with murine lung organoids (MiLO). MiLO generated from C57BL/6 mouse, widely used in environmental toxicology studies, were exposed to 10 μM Cd for 24 h and analyzed for extracellular matrix protein changes analogous to *in vivo* lung fibrosis. Immunostaining revealed increased collagen I (COL1A1) deposition and presence of alveolar type 2 cells (SPC) in Cd-treated MiLO (Figure 2d). To assess myofibroblast activation and invasiveness, MiLO were cultured on collagen-coated 96-well plates with Cd, and the percentage zone of invasion (ZOI%) was quantified, showing a significant increase in invasiveness (*p* < 0.0001, Figure 2e). Hydroxyproline assays performed on MiLO (n = 3) and Cd-exposed mouse lung tissue (day 21) confirmed comparable elevations in collagen content after normalizing for dry weight (Figure 2f). These results demonstrate that Cd exposure induces extracellular matrix remodeling and myofibroblast invasiveness in MiLO, mirroring pathological changes observed *in vivo*, thereby supporting the utility of MiLO as an *in vitro* platform for pulmonary toxicity studies.

### MiLO demonstrate oxidative stress comparable to mice lungs

Lipid peroxidation is a key marker of oxidative stress and contributes to the development of fibrotic responses in lung tissue (Ayala et al., 2014; Zhou et al., 2025). Malondialdehyde (MDA), a byproduct of lipid peroxidation, was significantly elevated in lung tissue from Cd-exposed mice (n = 3), indicating increased oxidative damage (Figure 2g). Similarly, murine lung organoids (MiLO) exposed to 10 µM Cd for 24 h showed a marked increase in MDA levels (*p* = 0.0001), confirming enhanced oxidative stress *in vitro* (Figure 2g). Additionally, superoxide dismutase (SOD) activity, an essential antioxidant defense enzyme, was significantly reduced in both Cd-exposed mice lung tissue (*p* = 0.0343) and Cd-treated MiLO (*p* < 0.0001) (Figure 2h). This decrease in SOD activity indicates impaired antioxidant capacity, thereby exacerbating oxidative stress. Together, these findings demonstrate that Cd exposure induces oxidative damage via increased lipid peroxidation and diminished antioxidant defenses, mechanisms known to promote myofibroblast activation and extracellular matrix remodeling in pulmonary fibrosis.

### Drug induced the pro-fibrotic response in MiLO

**FIGURE 3:**
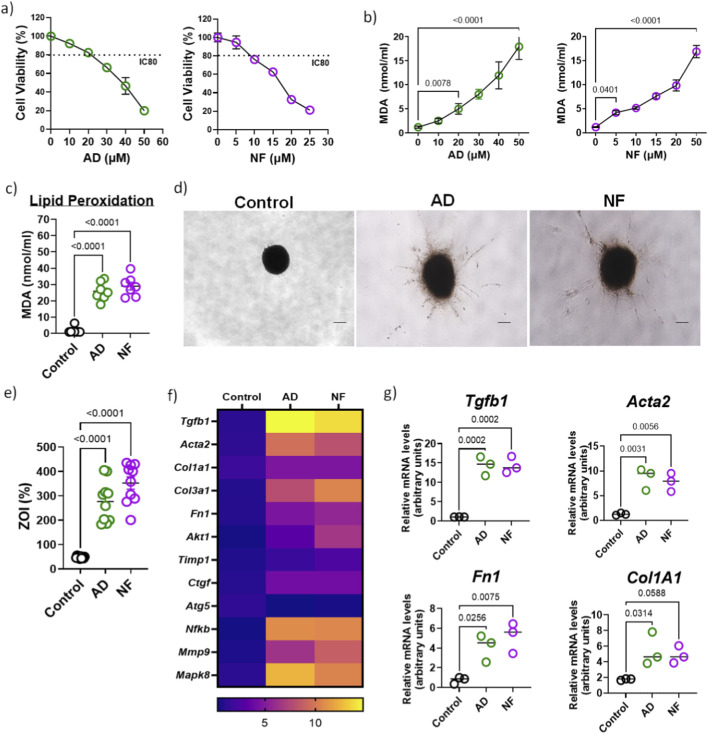
Therapeutic drugs induce the pro-fibrotic response in MiLO. **(a)** Cell viability assay for mice lung tissue derived cells was performed with increasing concentrations of amiodarone (AD; 0, 5, 10, 15, 20 and 50 µM) and nitrofurantoin (NF; 0, 5, 10, 15, 20 and 50 µM). **(b)** MDA assay for cells treated with increasing concentrations of amiodarone (AD; 0, 5, 10, 15, 20 and 50 µM) and **(d)** nitrofurantoin (NF; 0, 5, 10, 15, 20 and 50 µM). **(c)** Lipid peroxidation estimated using MDA assay for MiLO (n = 7) exposed to NF (5 µM) and AD (20 µM). **(d)** Invasion assay of MiLO exposed to NF (5 µM) and AD (20 µM) were observed using bright field microscopy. **(e)** Zone of invasion (%) was estimated for these MiLO. *p* < 0.0001. **(f)** Gene expression analysis shown with heat map of relative expression in MiLO exposed to AD and NF. **(g)** Individual mRNA expression analysis for *Tgfb1*, *Acta2*, *Fn1* and *Col1a1*.

Fibrosis invasion assays are essential for elucidating the progression and development of lung fibrosis. In this study, we evaluated two known pulmonary toxicants, nitrofurantoin (NF) (Cameron et al., 2000; Kaye et al., 2025; Suliman et al., 2023) and amiodarone (AD) (Terzo et al., 2020; Wolkove and Baltzan, 2009), both reported to induce pulmonary fibrosis as a side effect. Cell viability assays established 80% cell viability (or 20% cell inhibitory) concentrations for NF (5 µM) and AD (20 µM), which were used for subsequent experiments (Figure 3a). MDA levels, a marker of lipid peroxidation and oxidative stress, were significantly elevated in mice lung tissue derived cells exposed to increasing concentrations of these drugs (Figure 3b), correlating with increased MDA in MiLO exposed to NF (5 µM) and AD (20 µM) (Figure 3c). Both NF and AD triggered enhanced invasive behavior of MiLO cultured on collagen-coated plates, as demonstrated by increased invasion areas (Figures 3d,e). Consistent with their known fibrotic effects in human and murine lungs, NF and AD treatments induced upregulation of fibrosis markers in MiLO, including *Tgfb1*, fibronectin (*Fn1*), collagen 1 (*Col1a1*), and α-smooth muscle actin (*Acta2*) (n = 3, Figures 3f,g). These findings validate the use of MiLO for modeling drug-induced pulmonary fibrosis and highlight NF and AD as potent inducers of fibrotic responses via oxidative stress and myofibroblast activation. Additionally, increased expression of *Akt1*, *Ctgf*, *Nfkb1*, *Mmp9*, and *Mapk8* were observed, emphasizing the activation of signaling pathways associated with myofibroblast survival, matrix remodeling, inflammation, and stress responses (El-Tanbouly et al., 2017; Hsu et al., 2017; Larson-Casey et al., 2016; Nyp et al., 2014; Redente et al., 2021; Sinha et al., 2025). These findings support the use of MiLO for modeling drug-induced pulmonary fibrosis and highlight NF and AD as potent inducers of fibrotic responses via oxidative stress and myofibroblast activation.

## Discussion

IPF and ILDs present significant clinical challenges due to their progressive nature and complex etiologies, often involving environmental factors (Giménez et al., 2017; Gulati and Thannickal, 2019; Lee et al., 2025). Progressive pulmonary fibrosis arises from dynamic alveolar microenvironmental changes that drive epithelial cell loss and fibroblast/myofibroblast accumulation. These changes involve elevated profibrotic cytokines, growth factors, and chemokines, an imbalance favoring TIMPs over MMPs, and heightened oxidative stress (Thannickal et al., 2004). Environmental exposures to inhalable pollutants such as nanomaterials, carbon nanotubes, microplastics, and toxic metals like Cd have been identified as key contributors to lung injury and fibrotic remodeling (Baeza-Martínez et al., 2022; Brauer et al., 2016; Chen et al., 2022; Choong et al., 2014; Li et al., 2017; Zhang et al., 2024; Zhao et al., 2025). These agents penetrate deep into the alveolar spaces, inducing chronic inflammation, oxidative stress, and ECM accumulation that reinforces lung stiffness and functional decline. To accurately model these multifaceted processes, MiLO is presented as powerful *in vitro* system. Prepared from enzymatically digested mouse lung tissue and cultured in three-dimensional Matrigel, MiLO replicate lung tissue architecture and cellular heterogeneity, including alveolar type II epithelial cells (surfactant protein C positive), myofibroblasts (α-smooth muscle actin positive), endothelial cells (CD31 positive), macrophages (F4/80 positive), and airway basal progenitors (KRT5 positive). These cells situate within ECM proteins such as collagen type I and fibronectin-I, reflecting the lung microenvironment. Gene expression analyses of MiLO confirm the presence of fibrosis-associated markers, including *Tgfb1*, *Acta2*, collagens (*Col1a1* and *Col3a1*), *Ctgf*, *Lox2*, *Pdgfb*, and *Fn1*, validating MiLO as a biomimetic model for studying lung fibrogenesis (Bahram Yazdroudi and Malek, 2023; Giménez et al., 2017). Apoptosis and cell survival genes further ensure the physiological relevance of the model by confirming viable cellular populations amidst complex signaling dynamics.

Smoking, exposure to metals, including toxic metals such as Cd, and dust are strongly associated with an increased risk of developing ILDs, including idiopathic IPF and connective tissue disease-associated ILDs. Cigarette smoke is a significant contributor, with each cigarette containing approximately 2–3 µg of Cd, a heavy metal with a prolonged biological half-life of around 26 years in humans (Brauer et al., 2016; Caruso et al., 2013; Mannino et al., 2004), leading to harmful accumulation. To explore Cd’s role in fibrosis, mouse models exposed to doses simulating human lung exposure exhibited typical fibrotic markers such as increased α-smooth muscle actin (α-SMA), along with airway hyperresponsiveness and elevated hydroxyproline content (Li et al., 2021; Li et al., 2017). Critically, murine lung organoids (MiLO) exposed to physiologically relevant Cd concentrations recapitulated these pathological features, showing increased collagen I deposition and myofibroblast invasion within extracellular matrix scaffolds. This parallelism validates MiLO as an efficient, translational *in vitro* model for studying Cd-induced pulmonary fibrosis, offering mechanistic insights and reducing animal use. Further investigation of Cd toxicity, dose-dependent pulmonary fibrosis in mice, was demonstrated by increased myofibroblast activation and collagen accumulation confirmed through histology, functional airway resistance testing, and molecular assays. When comparing these *in vivo* findings with MiLO exposed to Cd, both systems exhibited comparable increases in extracellular matrix remodeling and fibroblast invasiveness, substantiated via invasion assays and hydroxyproline quantification. These results affirm MiLO as a physiologically relevant platform for pulmonary toxicity research.

In addition to environmental agents, pharmacological compounds such as nitrofurantoin and amiodarone (Kaye et al., 2025; Suliman et al., 2023; Terzo et al., 2020; Wolkove and Baltzan, 2009), both recognized for their pulmonary toxicity, induce comparable fibrotic responses in MiLO. Exposure to these drugs significantly increased oxidative stress markers and fibroblast invasiveness while upregulating profibrotic genes, including *Tgfb1*, *Acta2,* collagen subunits *Col1a1* and *Col3a1*, and fibronectin (*Fn1*) (Budin et al., 2022; Cameron et al., 2000; Chung et al., 2001; Giménez et al., 2017). Moreover, pro-survival, matrix remodeling, inflammatory, and stress response pathways were activated, as indicated by elevated *Akt1*, *Ctgf*, *Nfkb1*, *Mmp9*, and *Mapk8* expression (Giménez et al., 2017). These coordinated molecular changes reflect the multifactorial nature of drug-induced fibrosis (Chung et al., 2001; McCall et al., 2024; Mendez et al., 2005; Miwa et al., 2008; Schwaiblmair et al., 2012) and underscore the utility of MiLO for screening therapeutic interventions targeting oxidative damage and profibrotic signaling. Both NF and AD significantly increased levels of malondialdehyde (MDA), a key marker of lipid peroxidation (LPO), indicating elevated oxidative damage to cell membranes (Bast et al., 2010). Concurrently, this rise in MDA correlated with increased LPO activity in MiLO exposed to these drugs, highlighting the impairment of cellular integrity due to oxidative stress. MiLO represents a significant advancement in pulmonary toxicity testing due to their ability to accurately mimic the complex cellular architecture and microenvironment of lung tissue. Unlike traditional 2D cultures, MiLO provides a three-dimensional multicellular context where key parameters such as LPO (MDA levels) and SOD activity can be quantitatively measured. These oxidative stress markers offer sensitive and physiologically relevant endpoints for assessing drug- or environment-induced lung injury. Furthermore, the zone of invasion (ZOI) assay applied to MiLO enables precise quantification of fibroblast invasiveness (Li et al., 2021), a critical aspect of fibrotic remodeling, thereby linking biochemical oxidative stress with functional fibrogenic phenotypes.

These organoids can be used for evaluation of the therapeutic potential of drugs, study of signaling pathways of disease and the causes of exacerbation of disease and associated findings. Some of the limitations of this system may include the loss of evidence of systemic and physiological effects which can only be studied in a live animal model. While animal models have yielded critical insights into fibrotic mechanisms, they often fall short as they carry ethical and logistical limitations. MiLO generated from mouse lung tissue displays reproducible multicellular architecture and fibrosis-relevant gene expression profiles comparable to native lung, indicating model-level homogeneity across preparations, while preserving intra-organoid heterogeneity in epithelial, mesenchymal, endothelial, and immune compartments. The consistent induction of collagen deposition, myofibroblast invasion, oxidative stress, and profibrotic signaling in response to cadmium, nitrofurantoin, and amiodarone further demonstrates functionally homogeneous fibrotic responses arising from this heterogeneous, lung-like niche. The use of MiLO as a tissue-derived platform offers a transformative alternative to traditional animal models for pulmonary fibrosis research. MiLO bridges this gap by mimicking lung cellular heterogeneity, extracellular matrix interactions, and fibrogenic signaling pathways in a controllable, three-dimensional environment.

## Data Availability

The original contributions presented in the study are included in the article/Supplementary Material, further inquiries can be directed to the corresponding author.
