# Supplementary material for: Profibrotic predictive toxicology in the lung
Source: Front Pharmacol. 2026 Feb 18;17:1766054. doi: 10.3389/fphar.2026.1766054 (PMC12957118; doi:10.3389/fphar.2026.1766054)
Supplement: Supplementary file 1 [file Table1.pdf]

Table 1: List of genes analyzed in mice lung tissue and MiLO:

Gene	Official Full Name
Col1a1	Collagen Type I Alpha 1 Chain
Col3a1	Collagen Type III Alpha 1 Chain
Lox2	Lipoxygenase 2
Atg5	Autophagy related 5
Fn1	fibronectin 1
Acta2	actin alpha 2, smooth muscle
Src	Non-Receptor Tyrosine Kinase
Ctgf	connective tissue growth factor
Tgfb1	transforming growth factor beta 1
Nfkb	nuclear factor kappa B
Bax	BCL2 associated X
Fas	Fas cell surface death receptor
Casp3	caspase 3
Casp8	caspase 8
Mapk8	mitogen-activated protein kinase 8
Krt5	keratin 5
Akt1	Akt serine/threonine kinase 1
Mmp9	matrix metallopeptidase 9
Mmp13	matrix metallopeptidase 13
Pdgfb	platelet derived growth factor subunit B
Timp1	tissue inhibitor of metalloproteinase 1
Serpine2	serine (or cysteine) peptidase inhibito
Sftpc	surfactant protein C
Txnip	thioredoxin interacting protein
